# *Crocin* Acting as a Neuroprotective Agent against Methamphetamine-induced Neurodegeneration via CREB-BDNF Signaling Pathway

**DOI:** 10.22037/ijpr.2019.2393

**Published:** 2019

**Authors:** Shiva Mozaffari, Sanaz Ramezany Yasuj, Majid Motaghinejad, Manijeh Motevalian, Reyhaneh Kheiri

**Affiliations:** a *Department of Pharmaceutics, Faculty of Pharmacy, Tehran University of Medical Sciences, Tehran, Iran.*; b *Research Center for Addiction and Risky Behaviors (ReCARB), Psychiatric Center, Iran University of Medical Sciences, Tehran, Iran.*; c *Department of Pharmacology, School of Medicine, Iran University of Medical Sciences, Tehran, Iran.*; d *Department of Toxicology and Pharmacology, Faculty of Pharmacy, Tehran University of Medical Sciences, Tehran, Iran. *

**Keywords:** Methamphetamine, *Crocin*, Neurodegeneration, CREB, BDNF

## Abstract

Methamphetamine (METH) abuse causes neurodegeneration. Medicinal herb such as *crocin* has neuroprotective properties. The current study evaluates the role of CREB-BDNF signaling pathway in mediating the neuroprotective effects of *crocin* against METH-induced neurodegeneration in rats. Sixty adult male rats were divided randomly into group 1 and group 2 which received 0.7 mL/rat of normal saline and 10 mg/kg of METH intraperitoneally (i.p) respectively, and groups 3, 4, 5 and 6 which treated concurrently with METH (10 mg/kg) and *crocin *(10, 20, 40 and 80 mg/kg I.P respectively) for 21 days. Morris water maze (MWM) was used to evaluate cognitive activity. According to the critical role of hippocampus in cognitive behavior, the molecular and biochemical parts of our study were done in hippocampus and according to this, hippocampal neurodegenerative parameters and also CREB and BDNF levels were evaluated in isolated hippocampus. METH disturbed the learning, memory, and simultaneous treatment with various doses of *crocin* reduced the METH-induced cognition disturbances. In addition, METH treatment increased lipid peroxidation and the levels of oxidized form of glutathione (GSSG), interleukin 1 beta (IL-1β), tumor necrosis factor alpha (TNF-α), and Bax, while reducing reduced form of glutathione (GSH), Bcl-2, P-CREB, and BDNF levels in the hippocampus. METH also reduced the activity of superoxide dismutase (SOD), glutathione peroxidase (GPx), and glutathione reductase (GR) in the hippocampus. In contrast, *crocin* (40 and 80 mg/kg) attenuated METH-induced apoptosis, oxidative stress, and inflammation, while elevating P-CREB and BDNF levels. Thus, *crocin* confers neuroprotection against METH-induced neurodegeneration in hippocampus and this is probably through activation of P-CREB/BDNF signaling pathway.

## Introduction

Abuses of METH, as a neurostimulator agent, has been increased in recent years (1, 2). The consequences of chronic use of METH and its biochemical and behavioral effects remain unclear (3, 4). METH causes increase in release of dopamine, norepinephrine into synaptic terminals (4, 5). It causes hyper-stimulation of receptors in the acute phases and also down regulation of its receptor in chronic phase (4, 5). METH action, pharmacologically, is similar to cocaine and this similarity causes high potential of abuse and addiction (5). Chronic abuses of METH can induce behavioral changes such as anxiety and depression-like behavior and also cognition (learning and memory) impairment in rodent experimental models (6, 7). Experimental studies have confirmed the potential effect of METH in neurodegeneration of some areas of the brain such as the hippocampus, responsible for cognition and anxiety (7). Previous studies have demonstrated that METH abuse can lead to production of apoptotic proteins like Bax, caspase-3, 8 and 9 and therefore, it causes DNA fragmentation in some brain regions, such as hippocampus and amygdala (8, 9). METH and other neuro-stimulant compounds can cause inflammation , oxidative stress and mitochondrial dysfunction in brain cells, but it’s putative mechanism remains unknown (10, 11). Interestingly, METH-induced neurotoxicity appears to be more pronounced in some brain regions like hippocampus (CA1, CA2, CA3, and DG regions) and amygdala (11-13).

During recent years, using herbal/natural compounds with therapeutic probability have been amazingly increased. Natural flavonoids and their derivatives are being extensively considered as therapeutic agents against neurodegenerative diseases and some Neuro-disorders induced by drug abuse (14, 15). *Crocin* is a carotenoid chemical compound that is found in the flowers crocus and gardenia (16-18). *Crocin* is the ingredient which is primarily responsible for the color of saffron (17, 19 and 20). *Crocin* has been shown to be an antioxidant, and neural protective agent (19, 21 and 22). The antioxidant behavior of *crocin* is related to the sugar moiety in *crocin* molecule which has a vital role in its chemical reactivity (19). Also, *crocin* has possible antidepressant properties in mice and humans (23). 

It exerts biological effects through its antioxidant, anti-inflammatory, antiapoptotic, and immunomodulatory activities (23-25). *Crocin* treatment has shown to counteract oxidative stress by reducing lipid peroxidation and improving the activity of antioxidant enzymes like superoxide dismutase (SOD) and catalase in some neurodegenerative disorder (24, 26). Furthermore, chronic treatment with *crocin* reduces the alcohol-induced rise in TNF-α and IL-1β, levels (27). All these properties may contribute to therapeutic potential efficacy of *crocin* in neurodegenerative disorders of drug abusers, but it’s exact mechanism remains unclear (27-29). Cyclic AMP response element binding protein (CREB) is a chief transcription factor which is involved in regulation of genes associated with synaptic and survival of neurones, neuroprotection and neural plasticity such as Brain-derived neurotrophic factor (BDNF) (30, 31). BDNF is an important neurotrophic factor which primarily supports the growth and survival of neurons. It is highly expressed in some brain areas that are known to regulate cognition, emotions, and rewards (17, 32). It is suggested that *crocin* may protect hippocampal and frontal neurons against stress-induced damage via up- regulation of CREB and BDNF, but this concept was not approved definitely. Thus, we designed this study to assess whether *crocin* confers neuroprotection against METH-induced hippocampal damage, and to determine the role of P-CREB-BDNF signaling pathway in this protection.

## Experimental

*Animals*

Sixty adult Wistar male rats, weighing between 250–300 g, were purchased from lab house of Iran University of Medical Sciences. They were kept under controlled condition, room temperature (22 ± 0.5 °C) with 12-h light/dark cycle and had free access to food and water. Our experiment protocol was approved by the Committee of Research in Ethics by the Iran University of Medical Science (Research code: 98-1-37-14561).

**Table 1:** Effects of various doses of *crocin *on mitochondrial GSH and GSSG content in METH treated rats

**Group**	**Mean ± SEM GSH (nmol/mg protein)**	**GSSG (nmol/mg protein)**	**GSH/GSSG**
Control group	62.7 ± 4.1	0.98 ± 0.2	76
METH (10 mg/kg)	40.5 ± 4.1^a^	5.2 ± 1.1^a^	11^a^
METH + *Crocin *(10 mg/kg)	46.5 ± 3.4	4.9 ± 0.04	15.3
METH + *Crocin *(20 mg/kg)	50.4 ± 5	4.69 ± 0.14	17.04
METH + *Crocin *(40 mg/kg)	56.3 ± 4.3^b^	3.5 ± 0.12^b^	36.3^b^
METH + *Crocin *(80 mg/kg)	58.2 ± 2.8^b^	3.5 ± 0.10^b^	42.6^b^

All data are presented as mean ± SEM, n = 8.

a *P *< 0.001 *vs. *control group

b *P *< 0.001 *vs. *METH group. METH: Methamphetamine.

**Figure 1:**
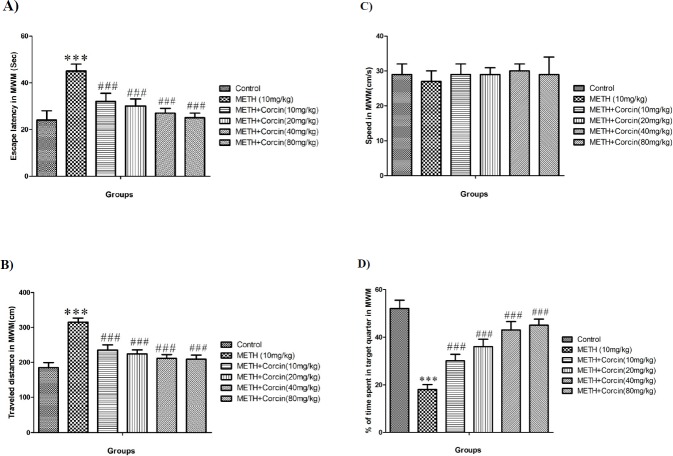
(A) Average of escape latency, (B) Average of traveled distance, (C) Average of swimming speed and (D) percentage of time spent in target quarter in probe trial in control group and groups treated with 10 mg/kg of METH and 10 mg/kg of METH in combination with *crocin *with doses of 10, 20, 40 and 80 mg/kg across all training days using Morris water maze (MWM) in rats.

All data are expressed as Mean ± SEM (n = 8).

***P < 0.001 *vs. *control.

###*P *< 0.001 *vs. *10 mg/kg of METH. METH: Methamphetamine.

**Figure 2:**
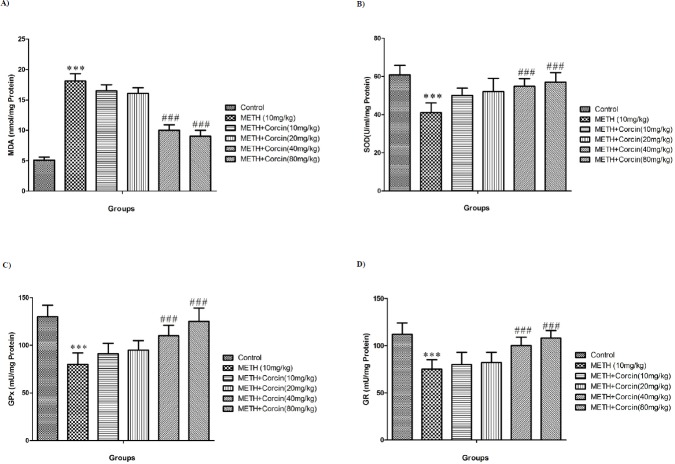
Effects of various doses of *crocin *(10, 20, 40 and 80 mg/kg) on METH-induced (A) lipid peroxidation, (B) SOD activity, (C) GPx activity and (D) GR activity in rat isolated hippocampus

All data are expressed as Mean ± SEM (n = 8).

***P < 0.001 *vs. *control.

**###** *P *< 0.001 *vs. *10 mg/kg of METH. METH: Methamphetamine.

**Figure 3:**
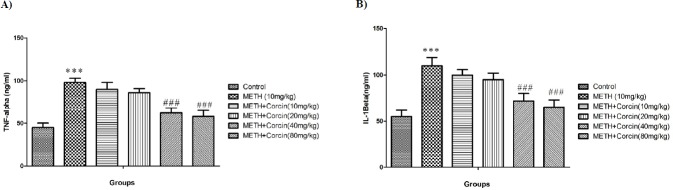
Effects of various doses of *crocin *(10, 20, 40 and 80 mg/kg) on METH-induced alteration in (A) TNF-α and IL-1β (B) level in rat isolated hippocampus

All data are expressed as Mean ± SEM (n = 8).

***P < 0.001 *vs. *control.

**###** *P *< 0.001 *vs. *10 mg/kg of METH. METH: Methamphetamine.

**Figure 4:**
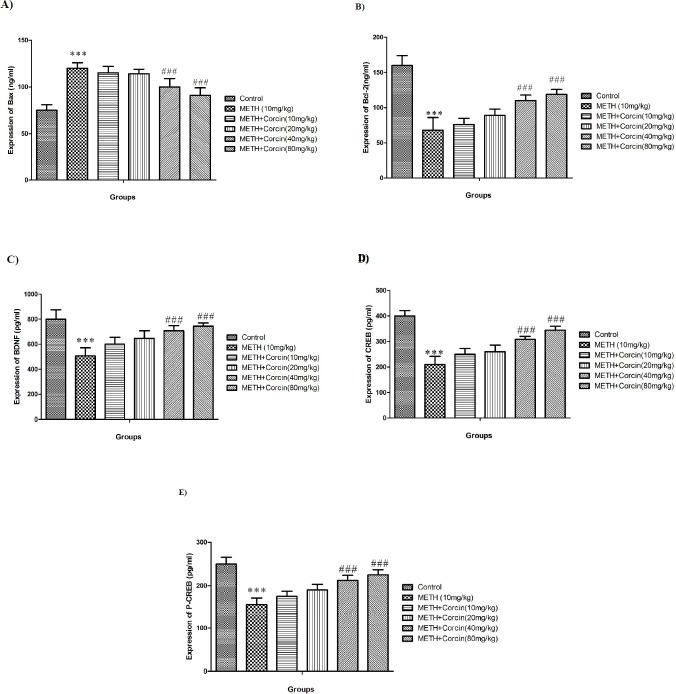
Effects of various doses of *crocin *(10, 20, 40 and 80 mg/kg) on METH-induced alterations in protein expression of (A) Bax, (B) Bcl-2, (C) BDNF, (D) total CREB and (E) phosphorylated CREB in rat isolated hippocampus. All data are expressed as Mean ± SEM (n = 8).

***P < 0.001 *vs. *control

**###** *P *< 0.001 *vs. *10 mg/kg of METH. METH: Methamphetamine.

*Drug*

*Crocin* and METH were purchased from Sigma-Aldrich (USA) and dissolved freshly in normal saline just before administration. 

*Experimental design*

Group 1 (control group) were administrated with normal saline (0.7 mL/rat, i.p) for 21 days and Group 2 (METH) received METH (10 mg/kg, i.p) for 21 days. 

Groups 3, 4, and 5 concurrently were treated by METH (10 mg/kg, i.p) and *crocin* with a dosage of 10, 20, 40, and 80 mg/kg, i.p., respectively for 21 days. It should be mentioned that in these groups the administration of *crocin* was done first and after one hour the METH was administrated. 

During the 17^th^ and 21^st^ day, morris water maze (MWM) task, a standard behavioral method for evaluation of learning and spatial memory, was performed. After 22^nd^ day, all animals were sacrificed and parameters for oxidative stress, inflammation, and apoptosis were evaluated in hippocampal tissues. Keeping in view the importance of CREB signaling and its product, BDNF, the effect of *crocin* on METH-induced disturbances in the CREB signaling pathway was studied in hippocampal tissues (33-35).

*Behavioral method*

*Morris water maze task (MWM)*

MWM apparatus includes a black colored circular tank, filled with water, 160 cm in diameter and 90 cm in height, which was fixed in the center of the experimental lab. This equipment was divided into four quadrants (North, East, West, and South) and was filled with water to the height of 50 cm. The operator stays in the North-East part of the room. A disk on the platform with 15 cm diameter, which was hidden, was located 1 cm beneath the surface of the water. In the first 4 days of the experiment, called training procedure, mentioned platform was randomly inserted persistently in one of the quarter. An automated infrared tracking system (CCTV B/W camera, SBC-300 (P), Samsung Electronics Co, Ltd, Korea) recorded the position of the experimented animal in the tank. The camera was mounted 2.4 m above the surface of the water (36, 37).

*A) Handling*

On the first day before the start of the experiment, all rats one by one were positioned on the tank that was filled with 40 ºC water, room temperature (25 ± 2 ºC) and the experimenter guided the rat for swimming and to reach to the quarter where platform was placed. In our experiment, the platform was situated on South-East quarter of a tank (36, 37).

*B) Training procedure*

Some discriminate landmarks (such as a distinguish picture, window, door, etc.) were placed in the extra maze in the room for spatial cues for learning about the platform’s position for animals. As mentioned above, the position of the platform was set up in the South-East quarter of the MWM tank with 25 cm distance from the edge of the tank, and 1 cm beneath the surface of the water. For evaluation of learning procedure, each rat experimented for 4 trials in a day for 4 days. Each animal was randomly located in from four quarters (North, East, West, and South) respectively. During the learning procedure if the rats found the platform within the 60 sec, the trial was automatically closed by a computer, but if they could not reach and find the platform within 60 sec the trial automat was stopped by computer. In learning experiment, two parameters were evaluated: 1) The time of escape latency characterized by time to find the hidden platform. 2) Traveled distance which was confirmed by the distance each animal spent to reach and find the hidden platform.

In memory assessment procedure, on the fifth day (probe day), the platform was removed and the animals were randomly terrified of the water from one of the above-mentioned directions (almost East) and the percentage of presence of the animals in the target quarter (South-East quarter) was recorded and calculated (36-40).

*Mitochondrial preparations*

The animals were anesthetized using sodium thiopental (50 mg/kg, i.p) and the hippocampus was isolated from each rat. The isolated tissues were homogenized in cold homogenization buffer (25 mM 4-morpholinepropanesulfonic acid, 400 mM sucrose, 4 mM magnesium chloride (MgCl_2_), 0.05 mM ethylene glycol tetraacetic acid (EGTA), pH 7.3) and the homogenized tissues were centrifuged at 450×g for 10 min. The supernatants obtained were re-centrifuged at 12000×g for 10 min. Finally, the sediments were re-suspended in homogenization buffer and stored at 0 °C. Total mitochondrial proteins in tissues were determined using a Dc protein assay kit (Bio-Rad), (California, USA). Briefly; Bradford reagent (1 part Bradford: 4 parts dH_2_O) was added to serial dilution series (0.1-1.0 mg/mL) of a known protein sample concentration; *e.g.*, bovine serum albumin (BSA), dissolved in homogenization buffer. These serial dilution series were prepared and used for providing a standard curve. On the other hand, 10, 15, 20, 25, and 30 μL of the protein extract (homogenized cell solutions) were added to multiple wells. Bradford reagent was also added to each well. The density of colors of all wells was read by the plate reader at 630 nm. Finally, by using the standard curve, protein quantity in the extracts was obtained. These homogenized cell solutions, containing mitochondria of hippocampal cells, were analyzed for the measurement of oxidative stress and inflammatory markers (34, 35, 41 and 42). 

*Measurement of oxidative stress parameters*

*Determination of lipid peroxidation*

For assessment of lipid peroxidation, malondialdehyde (MDA) - a natural by-product was assessed. Briefly, 100 μL of SDS lysis solution was added to wells containing (100 μL) of sample solution or MDA standard. After shaking and incubation of these wells, 250 μL of thiobarbituric acid (TBA) reagent was added to each well and incubated at 95 °C for 45-60 min. Next, the tubes were centrifuged at 1000×g for 15 min and 300 μL of n-Butanol was added to 300 μL of the supernatant. Then, the tubes were centrifuged for 5 min at 10,000×g. Finally, the absorbance was read at 532 nm and the results obtained were expressed as nmol/mg of protein (34, 35 and 41-44). 

*Determination of GSH (Glutathione) and GSSG (Glutathione disulfide) disulfide*

For measuring GSH (Glutathione) and GSSG (Glutathione disulfide) levels, 25 μL of the IX glutathione reductase solution and 25 μL of the IX NADPH solution were added to a 96-well plate containing a standard solution of glutathione or a sample of homogenized solution. Then, 50 μL of the IX Chromogen was added to each well and mixed vigorously. Finally, the absorbance was read at 405 nm for each GSSG/GSH standard and sample. Using the standard curve, the levels of GSSG/GSH were quantified and expressed as nmol/mg of protein (33, 44). 

*Determination of manganese superoxide dismutase (MnSOD) activity*

The previously described method was used to assess SOD activity (31, 33 and 44). SOD activity was measured using the following equation: SOD activity = {[(A blank 1 - A blank 3) – (A sample - A blank 2)] / (A blank 1 - A blank 3)} × 100. 

*Determination of glutathione peroxidase (GPx) activity*

GPx activity was assessed as previously described (31, 33 and 44). It was measured based on a change in absorbance [ΔA340/min] by the following equation:

ΔA340/min = A340nm (Start) – A340nm (Stop) /Reaction time (min), any change in the absorbance is directly proportional to GPx activity. 

GPx activity: ΔA340/min × Reaction volume (mL) × Dilution factor of the original sample/Extinction coefficient for NADPH at 340 nm × Volumes of the tested sample. The results were expressed as mU/mg protein (31, 33 and 44).

*Determination of glutathione reductase (GR) activity*

GR activity was assessed as described previously (31, 33 and 44). It was measured based on a change in absorbance [ΔA340/min] by the following equation:

ΔA340/min = A340nm (Start) – A340nm (Stop)/Reaction time (min), any change in the absorbance is directly proportional to GR activity. 

GR activity: ΔA340/min × Reaction volume (mL) × Dilution factor of the original sample/Extinction coefficient for NADPH at 340 nm × Volumes of the tested sample. The results were expressed as mU/mg protein (31, 33 and 44).

*Determination of protein expression alteration*

Concentrations (expression of protein) of brain-derived neurotrophic factor (BDNF), brain cyclic adenosine monophosphate (cAMP) (CREB) (total and phosphorylated), TNF-α, IL-1β, Bax, and Bcl-2 in cell lysate of hippocampal tissue, were measured by using a commercially available ELISA kit (Genzyme Diagnostics, Cambridge, U.S.A). Briefly, wells containing sheep anti-rat BDNF, CREB (total and phosphorylated), IL-1β, and TNF-α polyclonal antibody (Sigma Chemical Co., Poole, and Dorset, UK) were washed three times with washing buffer (0.5 M of Sodium chloride (NaCl), 2.5 mM sodium dihydrogen phosphate (NaH_2_PO_4_), 7.5 mM Na_2_HPO_4_, 0.1% Tween 20, pH 7.2). Then, 100 mL of 1% (w/v) ovalbumin (Sigma Chemical Co., Poole, Dorset, UK) solution was added to each well and incubated at 37 °C for 1 h. Following three washes, 100 ml of samples and standards were added to each well and incubated at 48 °C for 20 h. After three washes, 100 mL of the biotinylated sheep anti-rat IL-1β or TNF-α antibody (1:1000 dilutions in washing buffer containing 1% sheep serum, Sigma Chemical Co., Poole, and Dorset, UK) was added to each well. Next, after 1-hour incubation and three washes, 100 mL avidin-HRP (Dako Ltd, UK) (1:5000 dilution in wash buffer) was added to each well and the plate was incubated for 15 min. After washing three times, 100 mL of TMB substrate solution (Dako Ltd., UK) was added to each well and then incubated for 10 min at room temperature. Then, 100 mL of 1M H_2_SO_4_ was added and absorbance was read at 450 nm. The results were expressed as ng IL-1β/mL or TNF-α/mL or Bax and Bcl-2 of suspension of hippocampus tissues and about the BDNF and CREB (total and phosphorylated) were reported as pg/mL of suspension of hippocampus tissues (45-48).

*Statistical analysis*

The data were analyzed by GraphPad PRISM v.6 Software and averaged in every experimental group and expressed as Means ± Standard error of the means (SEM). Then, the differences between control and treatment groups were evaluated by ANOVA. To evaluate the severity of the behaviors, the differences between averages in each group were compared using the Tukey’s post-hoc at a significant level of *P* < 0.001. 

## Results

*Evaluation of escape latency and traveled distance during training days in the MWM*

METH (10 mg/kg) causes significant increase in escape latency (with F (5, 42) = 6.507) and traveled distance (with F (5, 42) = 12.46) during four days training in the MWM when compared to control groups (*P* < 0.001) (Figures 1A and 1B). While *crocin* in all doses inhibited METH-induced significant reduction in escape latency (with F (5, 42) = 6.507) and traveled distances (with F (5, 42) = 12.46) as opposed to METH (10 mg/kg) treated group (*P* < 0.001) (Figures 1A and 1B).

*Evaluation of swimming speed during training days*

The swimming speed was not altered during training trials in any of the animal groups (with F (5, 42) = 0.097) (Figure 1C).

*Evaluation of percentage in target quarter in probe trial*

METH, 10 mg/kg, causes significant decrease in the percentage of the presence of animals in target quarter (with F (5, 42) = 16.77) in comparison with control group (*P* < 0.001) (Figure 1D). Also, *crocin* in all doses used can significantly diminish METH induced decrease in presence of animals in target quarter (with F (5, 42) = 16.77) (Figure 1D).

*Effects of various doses of crocin on METH-induced GSH/GSSG alterations*

METH (10 mg/kg) treatment markedly reduced the mitochondrial GSH content, with F (5, 42) = 4.200, while increased the GSSG levels, with F (5, 42) = 11.20, in comparison to the control group (*P *< 0.001) (Table 1). Conversely, high doses of *crocin* (40 and 80 mg/kg) improved the GSH-content (with F (5, 42) = 4.200) and reduced the GSSG levels [F (5, 42) = 11.20] in METH-treated animals when compared to the positive control (*P *< 0.001) (Table 1).

*Effects of various doses of crocin on METH-induced alteration in oxidative stress parameters*

METH administration significantly increased the MDA levels (with F (5, 42) = 30.19) and reduced the SOD, GPx and GR activity with F (5, 42) = 1.80, F (5, 42) = 2.86 and F (5, 42) = 2.207, respectively when compared to the control group (*P *< 0.001) (Figures 2A-2D). Conversely**, **high doses of *crocin* (40 and 80 mg/kg) inhibit the METH-induced increase in MDA level (with F (5, 42) = 30.19) and decreases in SOD, GPx, and GR activity with F (5, 42) = 1.80, F (5, 42) = 2.86 and F (5, 42) = 2.207 respectively (*P *< 0.001) (Figures 2A-2D).

*Effects of various doses of crocin on METH-induced rise in inflammatory biomarkers*

METH, 10 mg/kg, causes significant elevation in level of IL-1β, with F (5, 42) = 8.36, and TNF-α, with F (5, 42) = 11.58, as compared to the control group (*P* < 0.001) (Figures 3A and 3B). Conversely, high doses of *crocin* (40 and 80 mg/kg) prevented the METH-induced rise in level of IL-1β, with F (5, 42) = 8.36, and TNF-α, with F (5, 42) = 11.58 when compared to METH only treated group (*P* < 0.001) (Figures 3A and 3B).

*Effects of various doses of crocin on METH-induced changes in Bax and Bcl-2 proteins level*

METH (10 mg/kg) treatment increased protein expression of Bax with F (5, 42) = 8.60 and reduced protein expression of Bcl-2, with F (5, 42) = 8.60, when compared to the control group (*P *< 0.001). Conversely, high doses of *crocin* (40 and 80 mg/kg) improved Bcl-2 expression while reduced Bax protein expression when compared to the positive controls (F (5, 42) = 8.60) (*P *< 0.001) (Figures 4A and 4B).

*Effects of various doses of crocin on METH-induced alteration in protein expression of both forms of CREB and BDNF*

METH (10 mg/kg) treatment markedly reduced the protein expression of BDNF and CREB (total and phosphorylated) with F (5, 42) = 3.53, F (5, 42) = 9.62, and F (5, 42) = 6.75, respectively when compared to control group (*P* < 0.001) (Figures 4C-4E). Conversely, in METH-dependent aniaml administartion of high doses of *crocin* (40 and 80 mg/kg) significantly improved the protein expression of BDNF and CREB (total and phosphorylated) [with F (5, 42) = 3.53, F (5, 42) = 9.62 and F (5, 42) = 6.75 respectively] when compared to the METH only treated group (*P *< 0.001) (Figures 4C-4E).

## Discussion

The results of current study demonstrated that various doses of *crocin* can ameliorate METH-induced neuro-apoptosis, oxidative stress, and inflammation in the rat hippocampus. In addition, it indicated that the protective role of *crocin* is mediated possibly via P-CREB /BDNF signaling pathway.

METH as a psycho-stimulant agent carries a high potential for abuse and addiction (8, 49). According to our study chronic administration of METH at a dose of 10 mg/kg can increase escape latency and traveled distance in MWM, this data suggested that METH administration can decrease learning activity. Also in probe day, METH administration could decrease percentage of presence in target quarter in MWM. These data suggested that chronic administration of METH can decrease spatial memory. These results confirm the results of previous study indicating chronic administration of METH decreased learning and memory in rats (8, 13). METH caused the release of dopamine, serotonin, and adrenaline in the brain and this releasing caused downregulation of the mentioned amine receptors and consequence of this phenomenon is cognition impairment (13, 50). According to our results, *crocin* at high doses (40 and 80 mg/kg) could alter the METH-induced cognition impairment. Many previous studies indicated that *crocin *and other similar herbal compound can improve learning and memory (25, 51). 

Our data indicated that administration of METH increases hippocampal MDA level, whereas, *crocin* treatment (10, 20, 40 and 80 mg/kg) attenuates METH-induced rise in lipid peroxidation in the brain. The inhibitory effect of *crocin* on MDA level was more significant at higher doses (40 and 80 mg/kg) compared to lower doses (10 and 20 mg/kg) used in this study. These results are similar to previous findings, which indicated METH-induced lipid peroxidation in the brain (8, 52). According to these data, it seems that part of the destructive effects of METH is mediated through mitochondrial dysfunction and probably *crocin* is somehow modulating this process (24, 53). Furthermore, it has been indicated by previous work that *crocin *exerts neuroprotective effects by inhibiting the formation of free radicals in neurodegenerative diseases such as Alzheimer (25), and the role of *crocin *as a scavenger for free radicals is well-evident in this type of disorder (24, 53). Our results indicated that METH (10 mg/kg) decreases mitochondrial GSH content while increasing GSSG level in the hippocampal tissues. Activation of glutathione reduced form (GSH) to the toxic oxidized form (GSSG) by METH is a key change that can start and activate neurodegenerative signals in the brain (54-57), and this mechanism causes harmful effect on glutathione cycle and consequently causes neural cell death (55, 57). Moreover, we found that various doses of* crocin*, especially 40 and 80 mg/kg, increase GSH content, while reducing GSSG level in animals with METH (10 mg/kg) consumption. These findings have been also reported already by previous studies indicating that *crocin*, by modulation of glutathione circle, can be therapeutically beneficial against neurodegenerative diseases as it promotes GSH formation (58, 59).

In our study, administration of METH decreased GPx, GR, and SOD activities in isolated hippocampal tissues which confirmed the previous studies report about METH abuse that diminishes antioxidant defenses which may result in neurodegeneration (13, 60). It has been shown that GR is the key enzyme which is responsible for converting the oxidized form of glutathione (GSH) to the reduced form (GSSG) (24). Thus, a METH-induced decrease in GR activity results in elevation of GSSG and reduction of GSH levels as observed in our results. Some novel reports showed that METH consumption causes mitochondrial dysfunction and lead to inhibition of antioxidant enzyme activity in multiple cells, and these properties caused METH-induced degenerative effects on brain cells such as hippocampus (8, 13 and 30). We detected that *crocin* treatment dose-dependently recovers the activity of antioxidant enzymes. *Crocin* by activating GR increases the conversion of GSSG to GSH and thus, protects the brain against METH-induced oxidative stress. Previous studies have also incdicated such anti-oxidative properties of *crocin* in neurodegenerative disorder (59, 61). In addition, our results confirmed the previous findings regarding the decrease in SOD activity following METH abuse (59). Consistent with previous studies, treatment by *crocin *was found to be effective in reversing the alcohol-induced reduction in SOD activity in the hippocampal tissues (59). 

We demonstrated that chronic METH administration significantly increases the level of pro-inflammatory cytokines like IL-β and TNF-α in the hippocampal tissue, whereas, *crocin* in high doses has a strong potential for suppressing METH-induced neuroinflammation in a dose-dependent manner. Our result is consistent with previous works which have reported the rise of pro-inflammatory cytokines following METH and other psychostimulant agents’ abuse. It has been proposed that METH-induced rise in inflammation is responsible for the neurodegenerative properties of METH (62). On the other hand, *crocin* has shown to have the therapeutic potential for management of neuroinflammation signaling cascades, thereby protecting the brain against inflammation and its damage (22).

In addition to oxidative stress and inflammation, this study confirms METH-induced apoptosis in the hippocampus. According to the current study, METH administration increased the level of an apoptotic protein, Bax, while decreasing an anti-apoptotic protein, Bcl-2. These data are inconsistent with previous works which have demonstrated that METH abuse can cause brain damage via activation of multiple apoptotic cascades (63, 64). On the other hand, our results demonstrated the anti-apoptotic effect of *crocin *against METH administration, as indicated by reducing Bax and improved Bcl-2 expressions in the hippocampus. Previous studies demonstrated that *crocin* treatment attenuates cleaved caspase-3 and production of Bax and nuclear condensation resulting from some neurodegenerative disorder and disease (65). 

The anti-inflammatory, anti-apoptotic, and anti-oxidative effects of *crocin* have been previously reported (22, 65), and were inconsistent with our work, but the involved signaling pathways remain unknown. In this regard, we evaluated the role of the P-CREB-BDNF signaling pathway. Our data demonstrated that METH administration ameliorates CREB (total and phosphorylated) and BDNF protein expression in the hippocampus. In contrast, *crocin* treatment in high doses enhanced CREB (total and phosphorylated) and BDNF protein expression. Thus, it can be speculated that *crocin* treatment restores P-CREB-BDNF signaling cascade and protects the brain against METH-induced neurotoxicity. The P-CREB, as a transcription factor, regulates over hundred target genes, especially BDNF, implicated in neuronal regeneration, development, survival, and excitability, addiction, depression and cognition (66). In addition, dysregulation of CREB transcriptional cascade has shown to induce oxidative stress, apoptosis, and neurodegeneration (66, 67). Many previous molecular studies verified that the phosphorylated form of CREB has the main role in many herbal and chemical neuroprotective possessions (67). According to numerous studies, P-CREB (activated form of CREB) causes the creation of BDNF, ligands of TrkB receptor. These works displayed that BDNF by stimulation of its own receptor, TrkB, can inhibits brain cell from degeneration and induces the existence of neurons (68). In the current study, it seems that reduction in P-CREB protein level, by METH, affects the mentioned cascade of BDNF/TrkB signaling pathway and triggers the neurodegeneration, apoptosis, inflammation and oxidative stress. While *crocin* administration inhibit this property of METH and can trigger cascade of P-CREB/BDNF/ TrkB. Consistent with our results, it has been shown that P-CREB-BDNF signaling pathway has been implicated in modification of several functions in brain such as learning, memory, mood balances, and reward mechanisms (68-71). 

## Conclusion

Taken together, for the first time, the results of the current study shows that *crocin* treatment, possibly via stimulation of P-CREB-BDNF signaling pathway, can decline METH-induced apoptosis, oxidative stress and inflammation and might possibly act as a neuroprotective agent against METH induced neurodegeneration. However, further studies regarding human dosage and toxicity are necessary. 
